# Small and Dim Target Detection via Lateral Inhibition Filtering and Artificial Bee Colony Based Selective Visual Attention

**DOI:** 10.1371/journal.pone.0072035

**Published:** 2013-08-21

**Authors:** Haibin Duan, Yimin Deng, Xiaohua Wang, Chunfang Xu

**Affiliations:** 1 State Key Laboratory of Virtual Reality Technology and Systems, School of Automation Science and Electrical Engineering, Beihang University (formerly Beijing University of Aeronautics and Astronautics), Beijing, P. R. China; 2 Science and Technology on Aircraft Control Laboratory, Beihang University, Beijing, P. R. China; UC Davis School of Medicine, United States of America

## Abstract

This paper proposed a novel bionic selective visual attention mechanism to quickly select regions that contain salient objects to reduce calculations. Firstly, lateral inhibition filtering, inspired by the limulus’ ommateum, is applied to filter low-frequency noises. After the filtering operation, we use Artificial Bee Colony (ABC) algorithm based selective visual attention mechanism to obtain the interested object to carry through the following recognition operation. In order to eliminate the camera motion influence, this paper adopted ABC algorithm, a new optimization method inspired by swarm intelligence, to calculate the motion salience map to integrate with conventional visual attention. To prove the feasibility and effectiveness of our method, several experiments were conducted. First the filtering results of lateral inhibition filter were shown to illustrate its noise reducing effect, then we applied the ABC algorithm to obtain the motion features of the image sequence. The ABC algorithm is proved to be more robust and effective through the comparison between ABC algorithm and popular Particle Swarm Optimization (PSO) algorithm. Except for the above results, we also compared the classic visual attention mechanism and our ABC algorithm based visual attention mechanism, and the experimental results of which further verified the effectiveness of our method.

## Introduction

Small and dim target detection in complicated background is a key technology and has found wide applications in such areas as remote sensing, distant early warning, space surveillance, aerospace, and so on [1], [2]. After atmospheric transmission and attenuation, the energy of long-distance imaging target is very weak when arriving at the imaging system, so the targets account for only one or a few pixels in the imaging plane. The shape of imaged target is fuzzy punctuate, and its features such as points, corner and edge are not obvious. As a result, the target is always submerged in the strong background noises, which makes the detection task a challenging one. What is more, detecting small objects in broad-area can be time consuming as the capabilities for image acquisition are growing rapidly. How to detect objects in large image data both quickly and reliably has become an increasingly pressing need.

In order to solve this problem, many methods have been proposed so far, such as dual-band infrared image fusion for dim target detection [3]; small target detect based on support vector machines in the wavelet domain [4]; target detect algorithm based on adaptive rectification filter [5]; target detection from the residual map between the original image and its background predicted image [6]; and small target detection algorithms in SAR image [7]. Nowadays, the selection of interest (ROI) based on visual attention in advance is more and more popular in the target detection area. Prior knowledge of the target accelerates target detection in visual search tasks [8].

Lateral inhibition mechanism is discovered and verified by Hartline and his research team when they carried out an electrophysiology experiment on the limulus’ vision [9]. This mechanism can enhance the contrast of sensory information, as well as reduce low-requency noises, which will do great help to detect dim object in cluttered background.

Visual attention mechanism is what Primates use to detect, in real time, conspicuous objects in cluttered visual environments. By using the sensitivity of the features including intensity, chromatic, orientation and motion, it could extract the interest information and orientate rapidly towards salient objects in complicated visual scene, which can accelerate the object detection process. In 1985, Koch and Ullman proposed the first visual attention mechanism model [10] based on the research of human cognitive psychology. This model first extracts several different characteristics, and then fuses them into a visual significant figure to simulate the process of finding interested region of human eye. On this basis, Itti put forward a new visual attention model suitable for processing natural image [11]. T.N. Mundhenk brought the contour integration concept into the model in 2005 [12]. C. Siagian presents a robotic positioning system based on visual attention mechanism in 2007 [13]. This system first exacts the interested area as the preliminary positioning assumption, and then based on this assumption to apply road signs matching for more precise positioning. L. Elazary and L. Itti proposed a new attention guide model for primary search and recognition [14], which was proved faster and more reliable. Visual attention mechanism has been more and more popular due to its broad applications, such as active information selection [15] and intelligent perception system [16], [17]. In our paper, we use it to accelerate the target detection process.

Positions of targets change irregular with the influence of camera motion and noise. Motion vector can be obtained as an important measurement characteristic in saliency map. In order to solve the problem of obtaining motion vector globally, some metaheuristic search algorithms are more potential as optimization algorithms have been proved to be apt to deal with function optimization problem. As a new optimization method, which is based on swarm intelligence and motivated by the intelligent behavior of honey bees, ABC algorithm has been proved to possess a better performance in function optimization problem, compared with genetic algorithm(GA), differential evolution (DE) and particle swarm optimization (PSO) [18], [19]. Therefore ABC algorithm is attracting more and more attention. ABC algorithm has been successfully used in unconstrained numerical optimization problems, artificial network training, image processing, path planning for UCAV(Unmanned Combat Aircraft Vehicle)and so on[18∼27]. A. Singh has proposed an ABC algorithm to solve the minimum spanning tree problem, and by comparing the experimental results with the ant colony algorithm, illustrates the superiority of ABC algorithm [28]. C. Xu applied ABC algorithm in the image processing are to show the advantage of avoiding local optimum of ABC algorithm [29]. In this paper we mainly use ABC algorithm to get the motion salience map of the visual image sequence.

To quickly select regions that contain salient objects to reduce calculations, a novel bionic selective visual attention mechanism is proposed in this paper, which is based on lateral inhibition filtering and ABC optimized motion feature. We attempt to adopt the lateral inhibition filter to illustrate its noise reducing effect, then the ABC algorithm is applied to obtain the motion feature of the image sequence. Combining our new feature channel with the typical model of visual attention, our proposed visual attention mechanism outperform others, hence to prove the feasibility and effectiveness of our model.

The remainder of this paper is organized as follows. Section 2 introduced the key principles and main procedure of our proposed method, and theoretical analysis is also conducted in this section. Series of experimental results and detailed contrast explanation are given in Section 3. Our concluding remarks and future work are contained also in the final section.

## Materials and Methods

**Figure 1:**
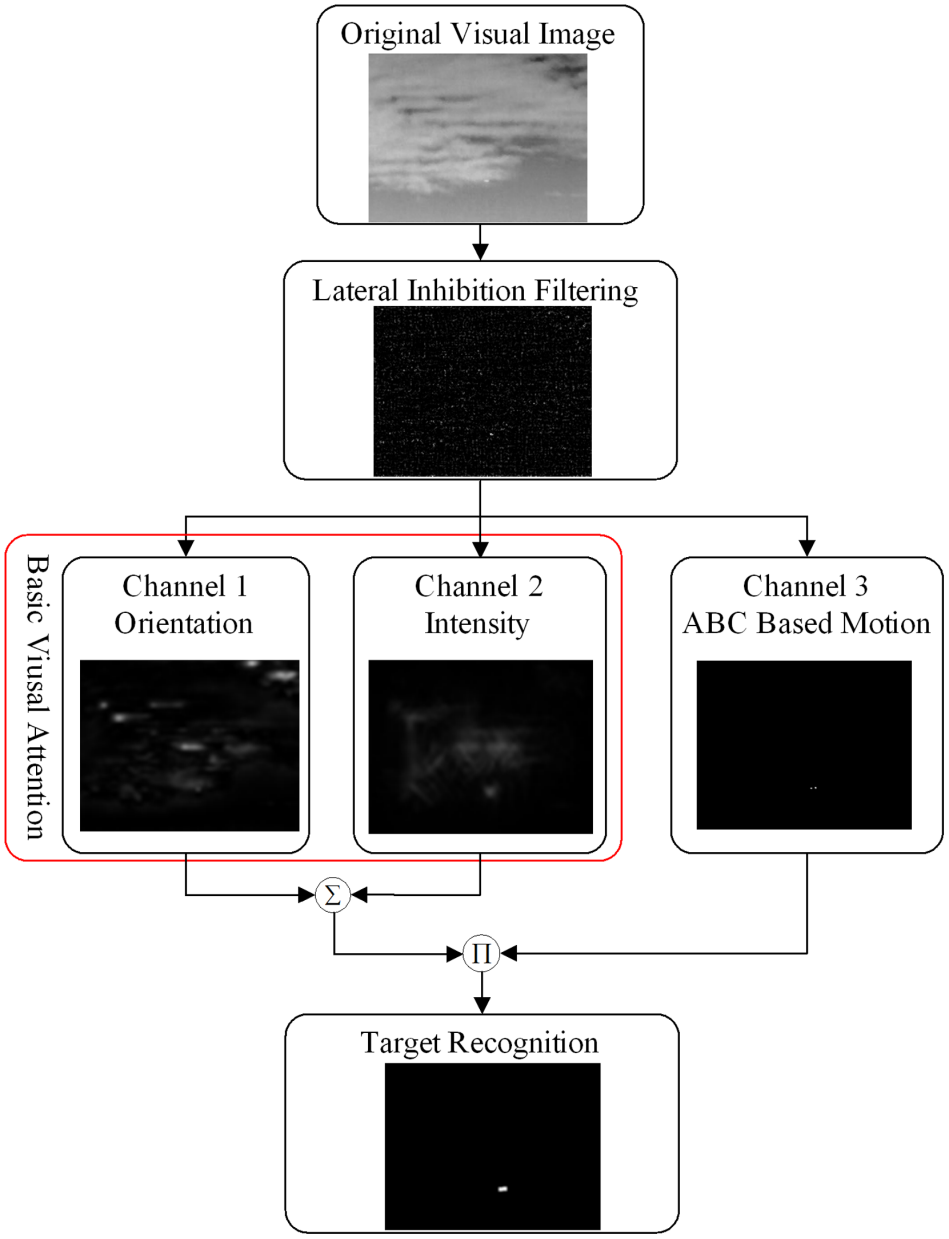
Our proposed target detection method.

In order to solve the small target detection problem, this paper proposed a novel bionic selective visual attention mechanism to quickly select regions that contain salient objects to reduce calculations. Fig. 1 shows the process of our proposed method. First lateral inhibition filtering is applied to reduce the low frequency noises, then our ABC based selective visual attention mechanism is used to get the motion feature of the image sequence and obtain the interested area to carry through the following recognition operation. If the object is proved to be not the target we aimed to detect, continue the visual attention operation to get the next interested object.

### Lateral Inhibition Filtering

The lateral inhibition mechanism is discovered and verified by Hartline and his research team when they carried out an electrophysiology experiment on the limulus’ vision [7]. They found that every microphthalmia of limulus’ ommateum is a receptor which is inhibited by its adjacent receptors, and the inhibited effect is mutual and spatially summed. According to their theory, every receptor is inhibited by its adjacent receptors, while it inhibits its adjacent receptors at the same time. The nearer the adjacent receptors are from each other, the more strongly they inhibit mutually.

In retinal image, the intensively excited receptors in illuminatingly light area inhibit the receptors in illuminatingly dark area more strongly than the latter to the former. Therefore, the contrast and the distortion of sensory information are enhanced. In this way, the important characters of vision scene and the intensity gradient in retinal image, namely the image’s edges, are both strengthened [30]. In this essay, this mechanism is applied to pre-processing of the original image to reduce low-frequency noises, which helps to detect the small and dim target in large field of view.

After a lot of electrophysiological experiments, Hartlein and his colleagues advanced the classical lateral inhibition model:
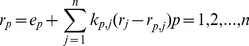
(1)


The function of the lateral inhibition enhancing the contrast of the image is verified in [30]. According to diverse classification criteria, lateral inhibition has many different modified models.

In order to introduce this mechanism to image processing, the classical lateral inhibition model is modified in two-dimensional and gray form. Gray value of the pixel (m, n) in image is given by:
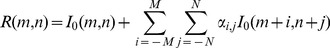
(2)where 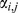
is the lateral inhibition coefficient of the pixel (*i*, *j*) to the central pixel, 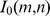 represents the original gray value of pixel 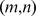, 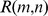
is the gray value of pixel 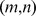 after being processed by lateral inhibition, and 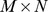
represents the receptive field.

The size of receptive field chosen in this essay is 5×5.Then the competing coefficient of the lateral inhibition network is:
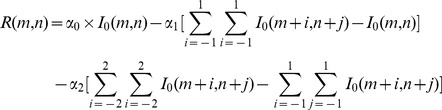
(3)where the lateral inhibition modulus satisfies: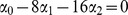. In this work, we choose 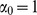,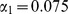,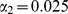. Then the modulus template *U* can be calculated with 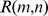
for convolution of input image as the following matrix:



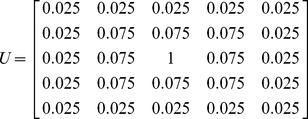
(4)After combining the modulus template *U* with 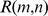, a new gray scales of the image can be obtained, which greatly reduced the low frequency noises.

### Static Features Based Visual Attention Mechanism

Visual Attention is an important psychological adjustment mechanism of human vision. Bottom-up visual attention is the process by which primates quickly select regions of an image that contain salient or conspicuous objects. The most important function of selective visual attention is to direct our gaze rapidly towards objects of interest in our visual environment, which can help accelerate the target detection.

The typical model of visual attention for static image was proposed by Koch and Ullman [8]. In this model, an image is analyzed along multiple low-level feature channels to give rise to multi-scale feature maps, which detect potentially interesting local spatial discontinuities using simulated center-surround neurons.

As the scenes we adopt are gray scales, the color characteristic in typical model of visual attention can not be used. Firstly, some features are extracted from the image. The features are one intensity feature(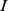) and four local orientation features (which is 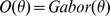) according to the angles 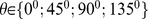.

Secondly, the multi-scale approach is aimed at detecting conspicuous features of different sizes to produce a set of images using imaging pyramids operator. In this paper, we obtain 9 scales, from scale 0(the original image) to scale 8(the image reduced by factor to 2^8^ = 256 in both the horizontal and vertical dimensions).

Six center-surround difference maps are then computed as point-to-point difference across pyramid scales, for combination of three center scales according to :
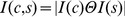
(5)

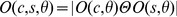
(6)where 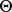 represents the center-surround operation, 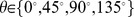, *c*∈{2,3,4},*δ*∈{3,4}, 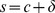. Therefore, for each feature, six feature maps are computed between scale2–5, 2–6, 3–6, 3–7, 4–7 and 4–8, yielding a total of 30 feature maps.

In this way, initially possibly very noisy feature maps are reduced to sparse representations of only those locations which strongly stand out from their surroundings. All feature maps finally contribute to the unique scalar saliency map, which represents visual conspicuity of each location in the visual field. The multi-scale maps are combined in a competitive way into a single feature conspicuity map in accordance by:
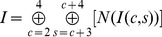
(7)

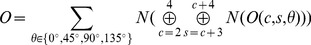
(8)where 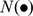
is a normalization function that simulates both intra-map competition and inter-map competition among the different scale maps. Then, using the same competitive map integration scheme, the feature maps are finally integrated together into a saliency map *S*.

After acquiring the final saliency map, the winner-take-all network is applied to get the attention area to carry through the following detection and recognition operations.

### Basic Principle of ABC Algorithm

ABC algorithm was firstly proposed by simulating the self-organization simulation model of honey bees [31]. In this model, although each bee only performs one single task, yet through a variety of information communication ways between bees, the entire colony can complete a number of complex works such as hives building, pollen harvest and so on. Then in 2003, Dušan Teodorović further introduced a bee colony optimization(BCO) algorithm [32]. After that Dervis Karaboga put forward an improved ABC algorithm, which we will use in our model [18], [19].

**Figure 2:**
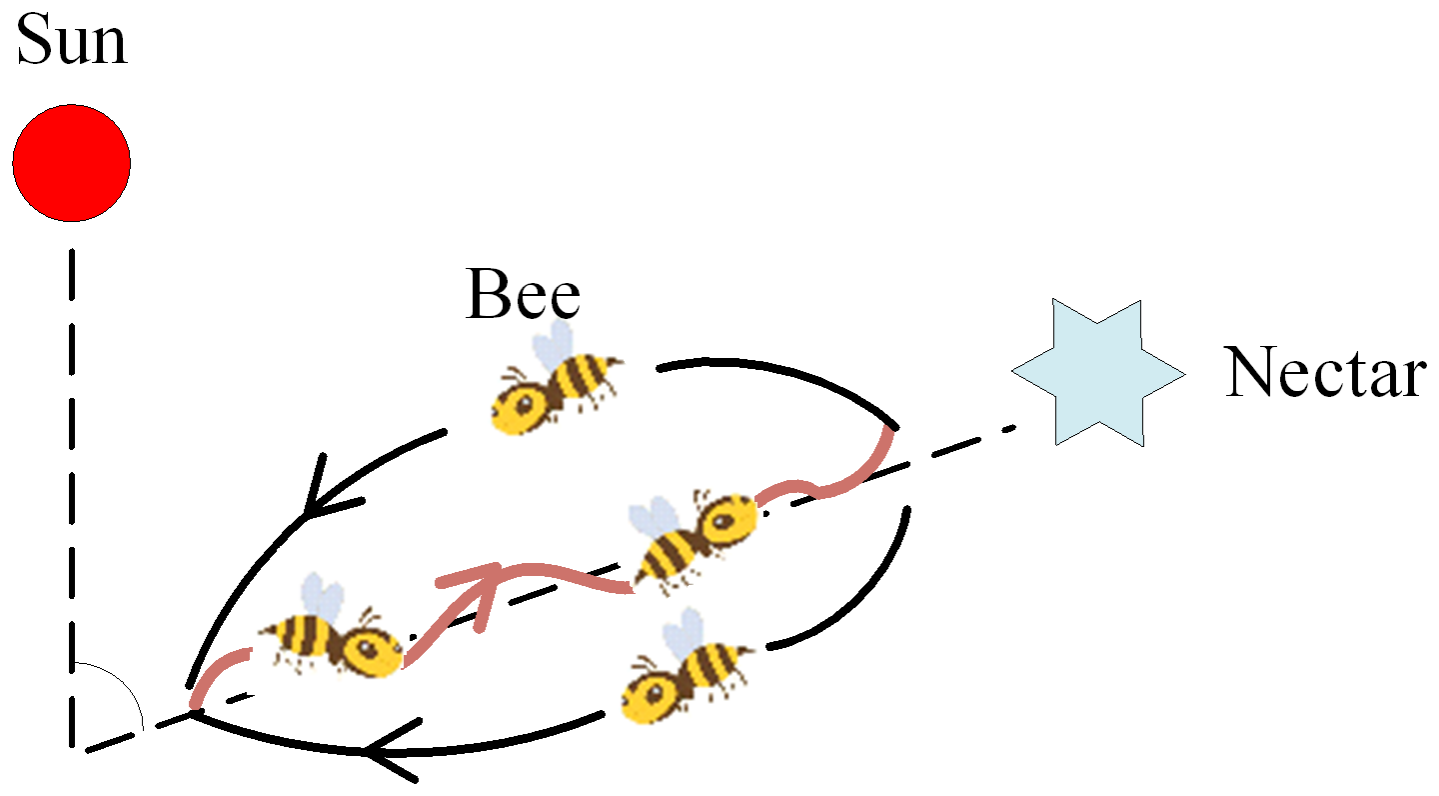
Waggle dance of honey bees.

Karlvon Frisch, a famous Nobel Prize winner, once found that in nature, although each bee only performs one single task, yet through a variety of information communication ways between bees such as waggle dance and special odor, the entire colony can always easily find food resources that produce relative high amount nectar, hence realize its self-organizing behavior [33]. In nature, the bees crawl along a straight line, and then turn left, moving as figure eight and swinging their belly. Such a dance is called waggle dance, and the angle between the gravity direction and the centre axis of the dance is exactly equal to the angle between the sun and food source (as shown in Figure 2). Through this kind of information exchanging and learning, the whole colony would always find relatively prominent nectar source.

In order to introduce the model of forage selection that leads to the emergence of collective intelligence of honey bee swarms. We need to define three essential components: food sources, unemployed foragers and employed foragers [18].

**Figure 3:**
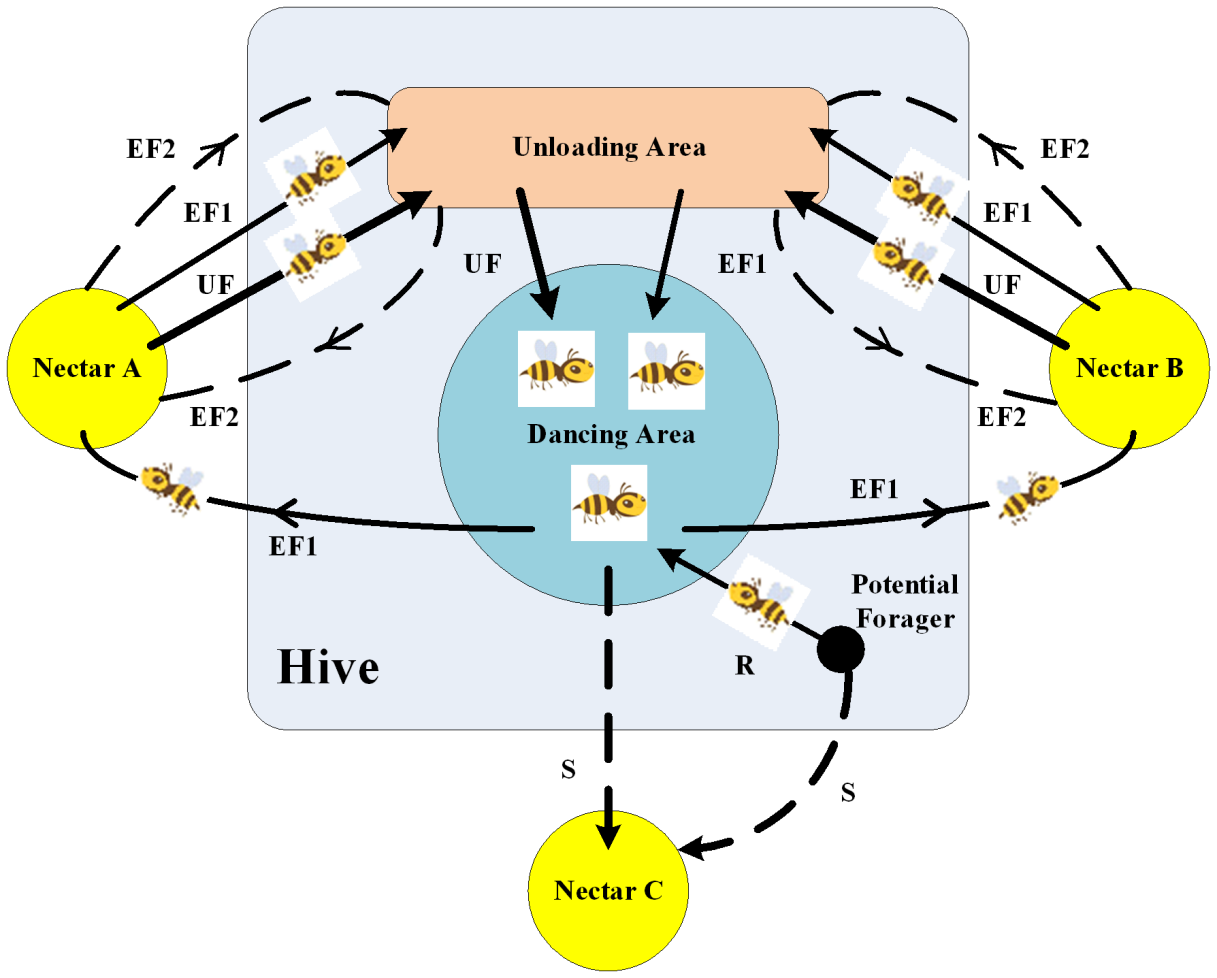
The behavior of honey bee foraging for nectar.

1. Food Sources (A and B in Fig. 3): For the sake of simplicity, the “profitability” of a food source can be represented with a single quantity [34], which corresponds to the similarity value in our target recognition problem.
2. Unemployed foragers (UF in Fig. 3): Unemployed foragers are continually looking out for a food source to exploit. There are two types of unemployed foragers: scouts(S in Fig. 3) and onlookers(R in Fig. 3).
3. Employed foragers (EF1 and EF2 in Fig. 3): They carry with them information about this particular source, the profitability of the source and share this information with a certain probability.

After the employed foraging bee loads a portion of nectar from the food source, it returns to the hive, unloads the nectar to the food area in the hive, and converts into any kind of bees (UF or EF) in accordance with the profit of the searched food sources.

In this paper, ABC algorithm is used to get the translation vector between images to obtain the movement characteristics.

### Mathematical Description of ABC Algorithm

Define *Ns* as the total number of bees, Ne as the colony size of the employed bees and *Nu* as the size of unemployed bees, which satisfy the equation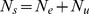. *D* is the dimension of individual solution vector, 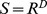 represents individual search space, and 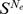
denotes the colony space of employed bees. An employed bee colony can be expressed by 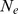 dimension vector 
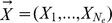
, where 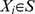 and 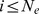. 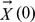 means the initial employed bee colony, while 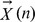 represents employed bee colony in the nth iteration. Denote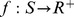 as the fitness function, and the standard ABC algorithm can be expressed as follows,

#### Step 1

Randomly initialize a set of feasible solutions 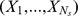, and the specific solution 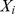 can be generated by
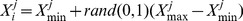
(9)where 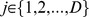 is the jth dimension of the solution vector. Calculate the fitness value of each solution vector respectively, and set the top Ne best solutions as the initial population of the employed bees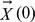.

#### Step 2

For an employed bee in the nth iteration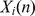, search new solutions in the neighborhood of the current position vector according to:
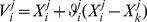
(10)where 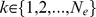,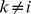, k and j are randomly generated. 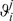 is a random number between −1 and 1.

Generally, this searching process is actually a random mapping from individual space to individual space, and this process can be denoted with 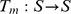, and its probability distribution is clearly only related to current position vector *Xi*(*n*), and has no relation with past location vectors as well as the iteration number *n*.

#### Step 3

Apply the greedy selection operator 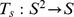 to choose the better solution between searched new vector Vi and the original vector Xi into the next generation. Its probability distribution can be described as:
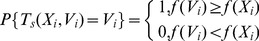
(11)


The greedy selection operator ensures that the population is able to retain the elite individual, and accordingly the evolution will not retreat. Obviously, the distribution of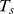 is has no relation with the iteration *n*.

#### Step 4

Each unemployed bee selects an employed bee from the colony according to their fitness values. The probability distribution of the selection operator 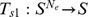
is :
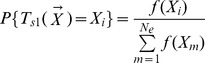
(12)


#### Step 5

The unemployed bee searches in the neighborhood of the selected employed bee’s position to find new solutions. The updated best fitness value can be denoted with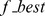, and the best solution parameters can be expressed with 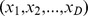.

#### Step 6

If the searching times surrounding an employed bee Bas exceeds a certain threshold Limit, but still could not find better solutions, then the location vector can be re-initialized randomly according to:
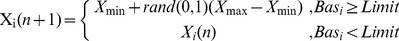
(13)


#### Step 7

If the iteration value is larger than the maximum number of the iteration (that is, *T*>*Tmax*), output the optimal fitness value 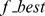 and correlative parameters 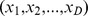. If not, go to Step 2.

Step 6 is a most prominent aspect making ABC algorithm different from other algorithms, which is designed to enhance the diversity of the population to prevent the population from trapping into the local optimum. Obviously, this step can improve the probability of finding the best solution efficiently, and make the ABC algorithm perform much better.

### Motion Features Acquisition Based on ABC Algorithm

**Figure 4:**
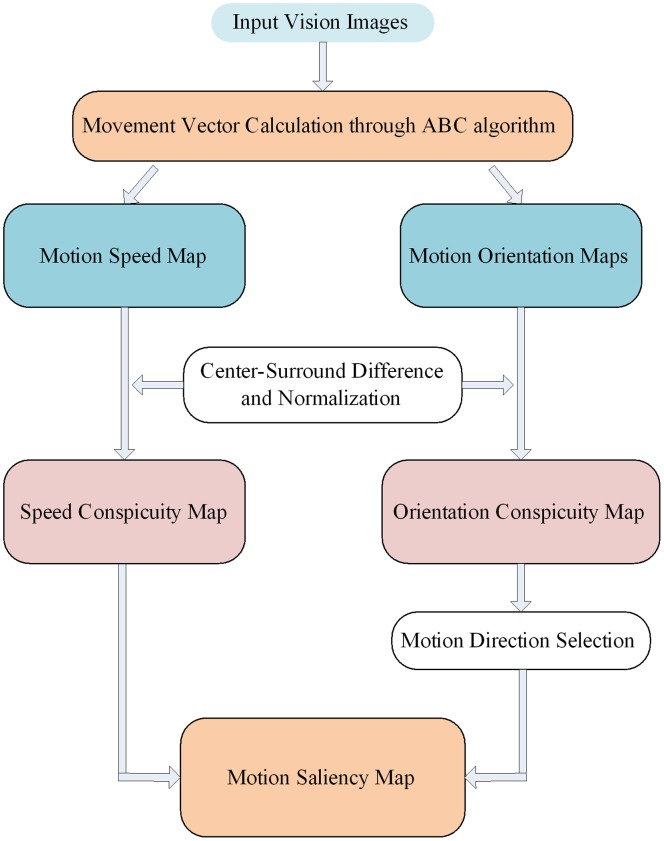
Architecture of our motion features acquisition process.

Generally, moving objects can always attract more attention than static objects, and the faster the object moves, the more attention it gets. Therefore, in our visual attention scheme, we presented movement characteristics including moving speed and direction to better detect and estimate the attention areas. Fig. 4 shows the architecture of our motion features acquisition process.

In the image matching process, we usually adopted general matching algorithms, which include optical flow vectors, character matching methods and so on. However, these algorithms would be influenced by the complex content of imagery, especially the computation of optical flow vectors. In this work, we use ABC algorithm to obtain the translation vectors 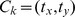
of every block. Here 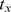
and 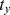 represent the horizontal and vertical translation respectively, the absolute value of which illuminate the intensity of the movement for each block.

**Figure 5:**
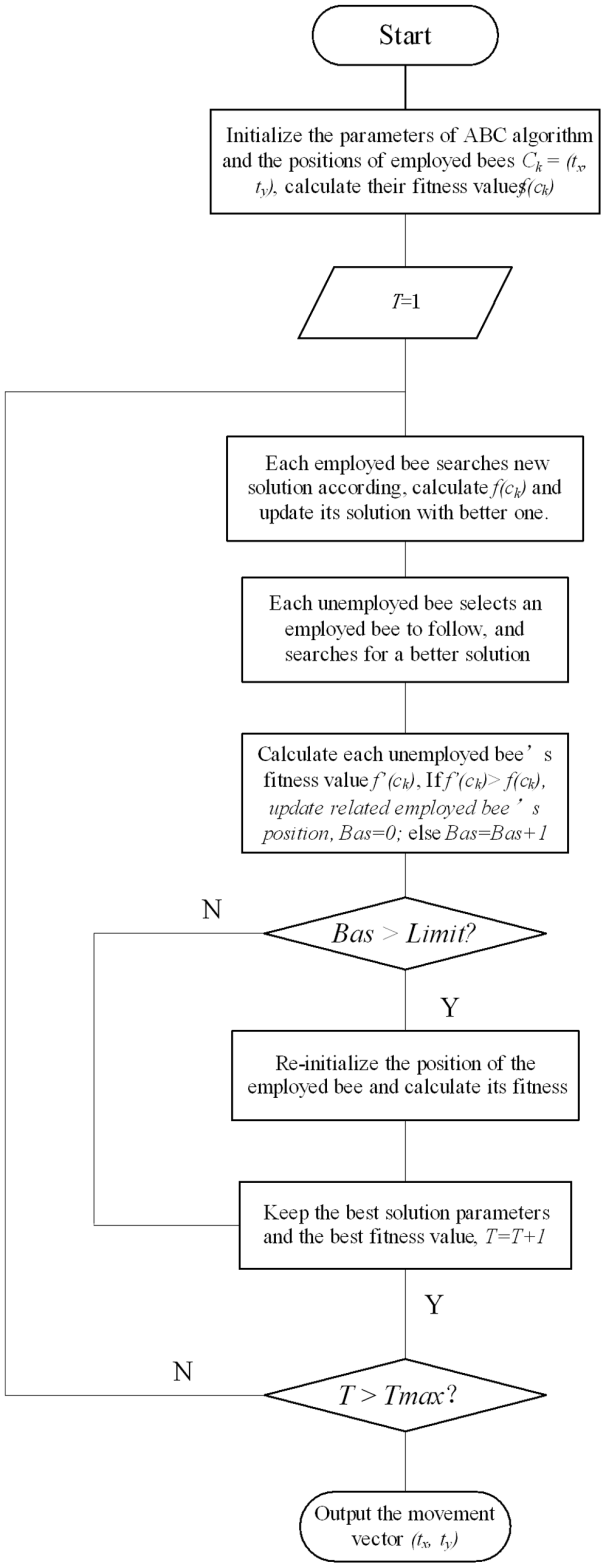
Procedure of ABC algorithm optimized movement vector acquisition.

In this paper, we define the matching criteria as :
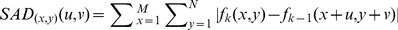
(14)where M*N is the size of the search window, and in this paper we define the size as 15*15. The translation vector, namely the motion vector is obtained when the SAD value finds its minimum.
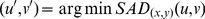
(15)where 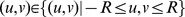, and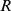 is the search range. The ABC algorithm based block matching method is described clearly in Fig. 5.

#### Step 1

Obtain the image, and convert it into grayscale format. For each pixel of the image, select an image block of fixed size surround the pixel.

#### Step 2

Initialize the parameters of artificial bee colony optimization algorithm, such as the population of the bee colony *Ns*, the number of employed bees *Ne* and the number of the unemployed bees *Nu*. A larger *Ns* will contribute to a larger possibility of finding the best solution of the problem, however, it also means an increased computing complexity of the algorithm. In general, we define *Ne = Nu*. Denote the largest searching times with Limit, current iterations with *T*, and the largest iterations with 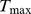. Initialize the population of geometric transformation parameters, which include the horizontal translation parameter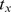 and the vertical translation parameter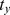. By considering these operators 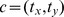, The goal is to find the optimal combination of parameters, which can provide the best fitness. Initialize the search time of each bee *Bas* = 0, and the starting iteration *T* = 1.

#### Step 3

According to the geometric parameters of the employed bees, calculate their similarity values respectively based on the defined similarity function. When the fitness function 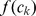 achieves its minimum value, we find the best solution to match the target image.

#### Step 4

The employed bees search around their current positions to find new solutions, and update their positions if the new fitness value is lower than the original value.

#### Step 5

The unemployed bees apply the roulette selection method to choose the bee individual that possesses a relatively good fitness value as the leading bee, according to the calculated fitness results of employed bees.

Each recruited unemployed bee continues to search new solutions just around the leading bee’s solution space, and calculate their fitness values. If the value of the new solution is better than the original value, the unemployed bee converts into an employed bee, which means that update the positions of the employed bees, and continue exploring with *Bas* re-initialized as 0, or else, keep searching around, and its *Bas* value plus one.

#### Step 6

If the search times *Bas* is larger than certain threshold *Limit*, the employed bee gives up the solution, and re-search the new food resources, which is realized by re-initializing the geometric parameters and calculating the fitness value.

#### Step 7

Store the best solution parameters and the best fitness value.

#### Step 8

If *T<T*max, go to Step 4. Otherwise, output the optimal parameters and optimal fitness value.

After getting the translation vectors of each block, we calculate the movement distance of each pixel in the image, generating the movement speed image *Ms* with the equation:
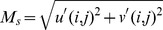
(16)


Except for this, we can also calculate 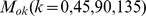 which denote the movement speed on four directions.
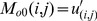
(17)

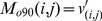
(18)

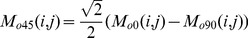
(19)

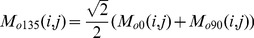
(20)


Through the center-surround operator, we calculate the salience maps according to:
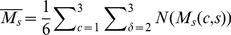
(21)

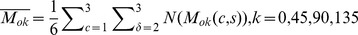
(22)


Choose the movement orientation salience map, the direction of which has the largest contrast, to fuse with the velocity salience map, in order to get the final motion salience map *S*.

## Results

In order to validate the performance of our proposed algorithm, experiments for small and dim target detection are performed on infrared images with sky-cloud.

**Figure 6:**
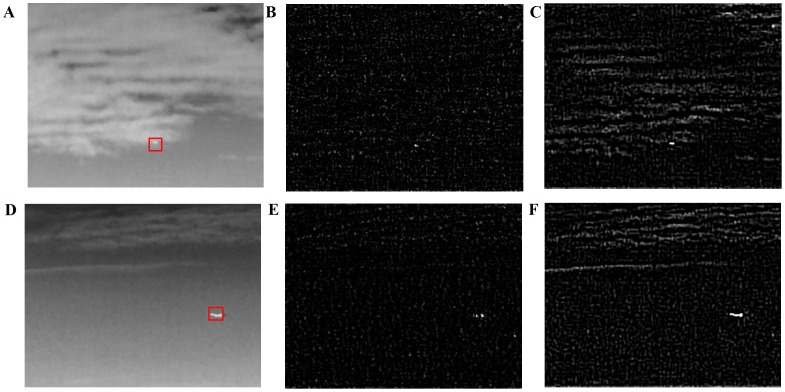
Experimental results. Two set of experimental results in different scenes. (A,D) The original scene.(B,E) Image after lateral inhibition filtering. (C,F) Image after high-pass filter.

According to our Filtering designed in Section II, we conducted several experiments, the results of which are shown in Fig. 6. The key details were strengthened to get the key target information, and the unneeded noises were eliminated, which proves the feasibility of the lateral inhibition filter. Meanwhile, we compare our algorithm with high-pass filter method. For fair comparison, we adopt a common evaluation indicator called Signal-to-Clutter Ratio (SCR) Gain [2], which reflects the amplification of target signals relative to backgrounds after and before processing. For two scenes in Fig. 6A and Fig. 6D, SCR Gain values of lateral inhibition filtering method (see Fig. 6B,E) are 6.3034 and 6.2606,respectively, while the corresponding values of high-pass filter method (see Fig. 6C,F) are 3.5660 and 3.6947. The results shows that lateral inhibition filtering method actually outperforms in small and dim target detection.

**Figure 7:**
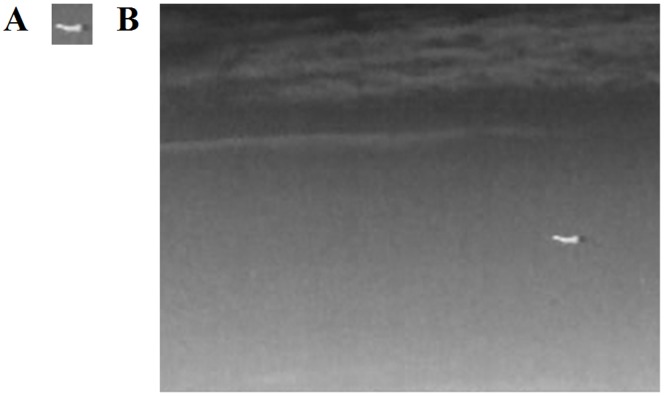
Test Image. (A) Template image. (B) Scene image.

To validate the performance of translation vector acquisition based on ABC algorithm, first we conduct an image matching test based on ABC algorithm and PSO algorithm. The images we used here are shown in Fig. 7. We calculate the translation parameters of the template image effectively using our proposed ABC algorithm. In order to compare the feasibility and effectiveness of our proposed algorithm, we compare our algorithm with another popular optimization algorithm PSO (Particle Swarm Optimization). The parameters of the two algorithms are as follows: Tmax = 30, Ne = Nu = 20, D = 2, Limit = 50 for ABC algorithm; Tmax = 30, N = 20 for PSO algorithm.

**Figure 8:**
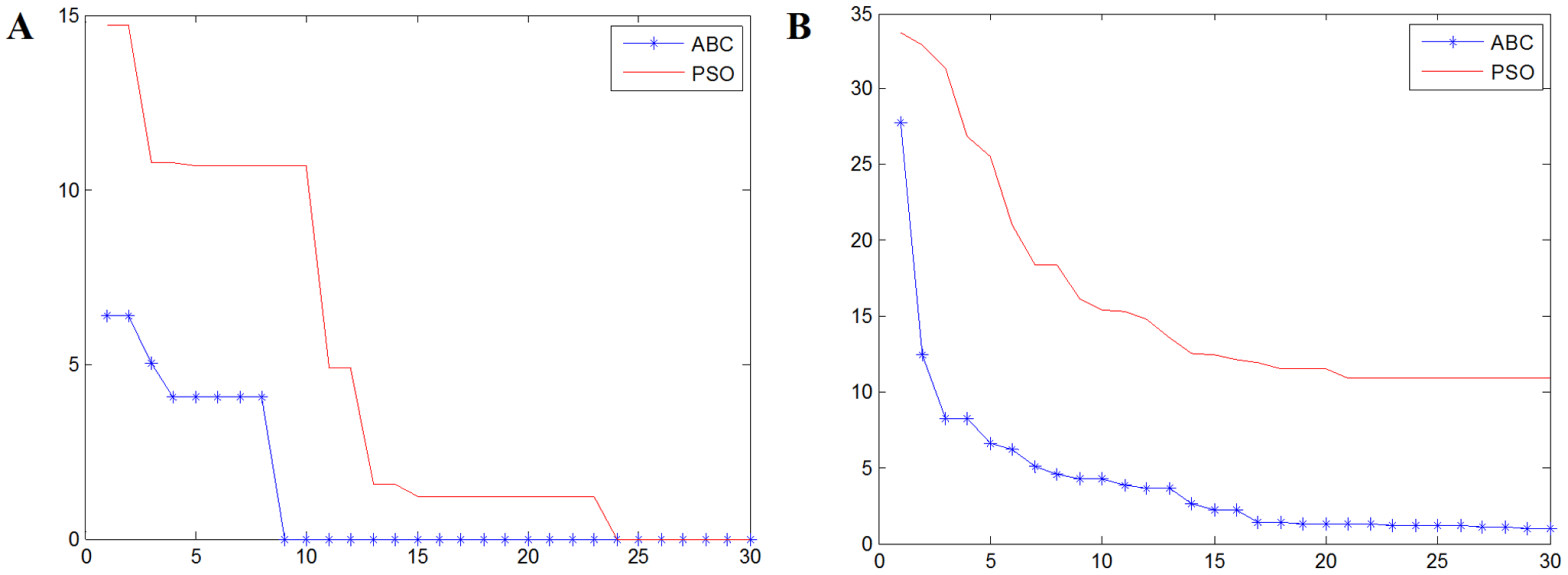
The comparison results of the two algorithm. (A) One-time evolution curve. (B) Average evolution curve.

We conduct this experiment for 10 times to get the average performance of the two algorithms, the results of which are shown in Fig. 8. In Fig. 8A, the ABC algorithm based result can find the global best effectively in the 9th iteration. In Fig. 8B the average fitness of ABC algorithm is lower than the PSO algorithm in each iteration, while the simulation result based on PSO algorithm is apparently apt to be trapped into local best, which proves the robustness and superiority of ABC algorithm compared with PSO algorithm. All in all, the experimental results above clearly show the feasibility and effectiveness of our ABC based algorithm compared with the popular PSO algorithm.

**Figure 9:**
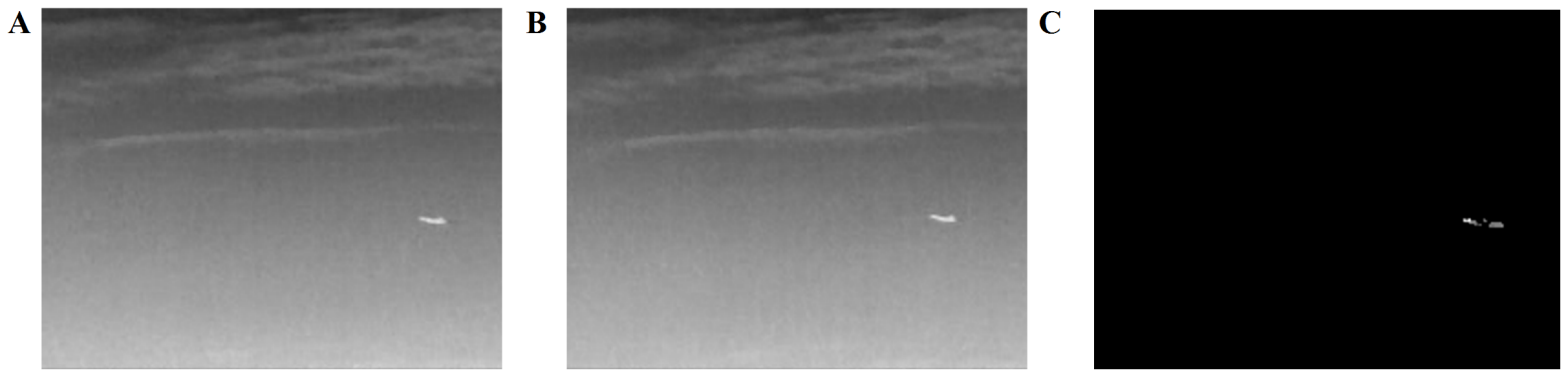
Movement salience map based on ABC algorithm. (A, B) Two frames. (C) Overall movement salience map.

**Figure 10:**
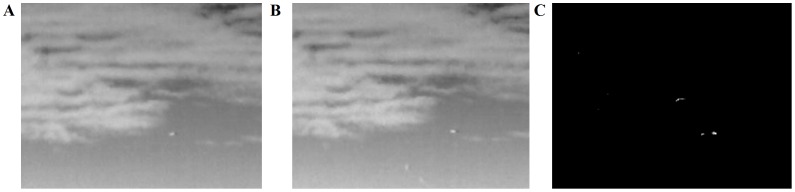
Another set of experiments. (A, B) Two frames. (C) Overall movement salience map based on ABC algorithm.

After applying ABC algorithm to get the motion features, we established our visual attention based on obtained motion features and conduct the experiment, the results of which are shown as follows. Fig. 9 and Fig. 10 give the movement salience map of an image sequence with varying cloud background, which separate the moving object significantly and quickly.

**Figure 11:**
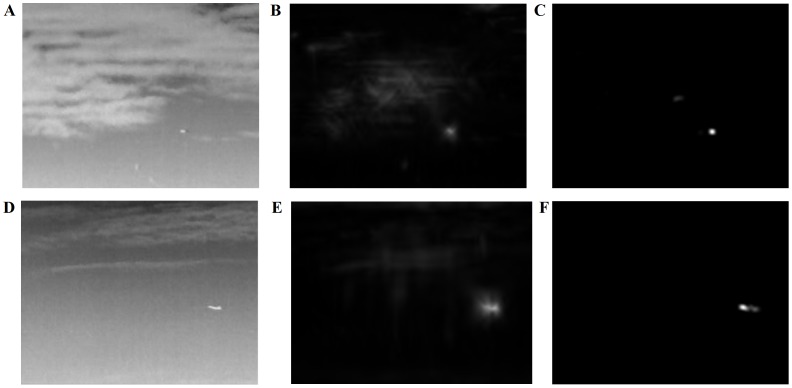
Experimental results. Two sets of experimental results in different scenes. (A,D) The original scene.(B,E) Overall salience map without movement factor. (C,F) Overall salience map with movement factor.

Fig. 11 shows that when we do not considering the motion features of the scene, some salient position lies in the trees of the background, hence could not focus quickly and effectively to the moving target. And after considering the motion features, the small and dim target can be effectively and quickly be highlighted as a significant target, which greatly accelerated the target detection and recognition.

**Figure 12:**
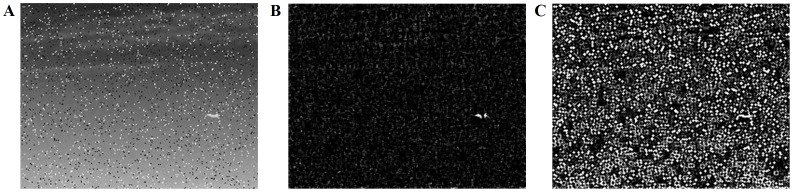
Comparison experimental results in the scene with strong clutters and noise. (A) The original scene.(B) Image after lateral inhibition filtering. (C) Image after high-pass filter.

**Figure 13:**
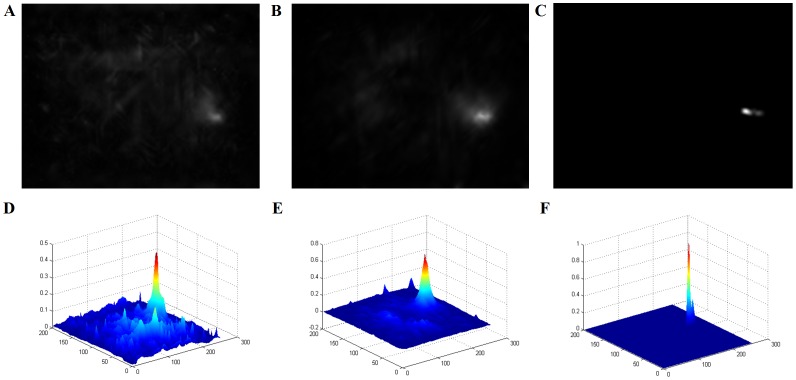
Experimental results of saliency-based target detection for the same scene in Fig. 12. (A) Overall salience map without lateral inhibition filtering and movement factor. (B) Overall salience map with lateral inhibition filtering. (C) Overall salience map with movement factor. (D-F) Energy distribution under mesh graph of the corresponding salience map from (A) to (C), respectively.

To further validate the performance and robustness of our proposed algorithm for small and dim target detection, we conduct several experiments in more challenging scenes, the results of which can be shown in Fig. 12 and Fig. 13. In the scene with strong clutters and noise, lateral inhibition filtering method obtains better performance than high-pass filter. For the same noisy scene, small and dim target is detected with our proposed saliency-based method From the comparison results of the salience map and corresponding energy distribution, we conclude that the object can be extracted effectively and exactly.

## Discussion

This paper proposed a novel bionic selective visual attention mechanism to quickly select regions that contain salient objects to reduce calculations. First, lateral inhibition filter is applied to filter low-frequency noises. After the filtering operation, we use ABC algorithm based selective visual attention mechanism to obtain the interested object to carry through the following recognition operation. To prove the feasibility and effectiveness of our method, several experiments were conducted.

The experimental results show that lateral inhibition filtering is effective to reduce the unneeded noises and highlight those of great significance, which can help detect target in cluttered background. The ABC algorithm is proved to be more robust and effective through the image matching comparison between ABC algorithm and popular PSO algorithm. And from the comparison between classic visual attention mechanism and our ABC algorithm based visual attention mechanism, our ABC algorithm based visual attention mechanism can extract reliable motion features form several adjacent frames effectively. Therefore our algorithm can put much more attention on the small and dim moving target and separate the moving targets from the background quickly for the following recognition process.
